# Comparative Analysis of Compatibility Influence on Invigorating Blood Circulation for Combined Use of Panax Notoginseng Saponins and Aspirin Using Metabolomics Approach

**DOI:** 10.3389/fphar.2021.544002

**Published:** 2021-04-30

**Authors:** Zongxi Sun, Huichao Wu, Yali Wu, Chenglong Wang, Yu Wang, Shaonan Hu, Shouying Du

**Affiliations:** ^1^ School of Chinese Materia Medica, Beijing University of Chinese Medicine, Beijing, China; ^2^ Institute of Ethnic Medicine, Guangxi International Zhuang Medicine Hospital, Nanning, China; ^3^ School of Pharmacy, Guangxi University of Chinese Medicine, Nanning, China; ^4^ Department of Pharmacy, The First Affiliated Hospital of Henan University of Chinese Medicine, Zhengzhou, China

**Keywords:** Panax notoginseng saponins, aspirin, metabolomics, blood stasis, herb-drug interactions

## Abstract

The combined use of Panax notoginseng saponins (PNS)–based drugs and aspirin (ASA) to combat vascular diseases has achieved good clinical results. In this study, the superior efficacy was observed *via* the combined use of PNS and ASA on acute blood stasis rats, and untargeted metabolomics was performed to holistically investigate the therapeutic effects of coupling application and its regulatory mechanisms. The combined use of PNS and ASA exhibited better improvement effects when reducing the evaluated hemorheological indicators (whole blood viscosity, plasma viscosity, platelet aggregation, and fibrinogen content) in the blood stasis rats vs. single use of PNS or ASA at the same dose. The combined use of both drugs was the most effective application method, as shown by the relative distance in partial least-squares discriminant analysis score plots. Twelve metabolites associated with blood stasis were screened as potential biomarkers and were mainly involved in amino acid metabolism, lipid metabolism, and energy metabolism. After coherently treated with PNS and ASA, the altered metabolites could be partially adjusted to be closer to normal levels than single use. The collective results revealed that PNS could cooperate with ASA to treat blood stasis and provided a scientific explanation for the superior efficacy of their combined use.

## Introduction

Blood stasis is a primary pathological concept of traditional Chinese medicine (TCM). It is considered an important factor in relation to many vascular diseases including stroke, angina pectoris, and acute myocardial infarction (Wang et al., 2014), and many such events could be effectively improved by eliminating blood stasis (Xiong, 2015; Ma et al., 2017). In TCM, one popular way to treat blood stasis is promoting blood circulation with medicinal herbs. Medicinal herbs were the major agents used for primary health care for many centuries before the advent of modern medicine (Fasinu et al., 2012). They are still applied with high frequency and continue to increase both in developing and developed countries (Murray et al., 2016). Herb–drug combined therapy is a common therapeutic strategy due to its therapeutic benefits (Sun et al., 2018a). Herbal products contain a large number of bioactive ingredients, some of which can interact with synthetic drugs. Considering the inevitable interaction between herbal and synthetic medicines, the reasonable herb–drug combined use can improve the therapeutic effect or reduce the toxicity, while the unreasonable application can produce toxicity or adverse reactions (Borrelli and Izzo, 2009; Brantley et al., 2014).

**FIGURE 1:**
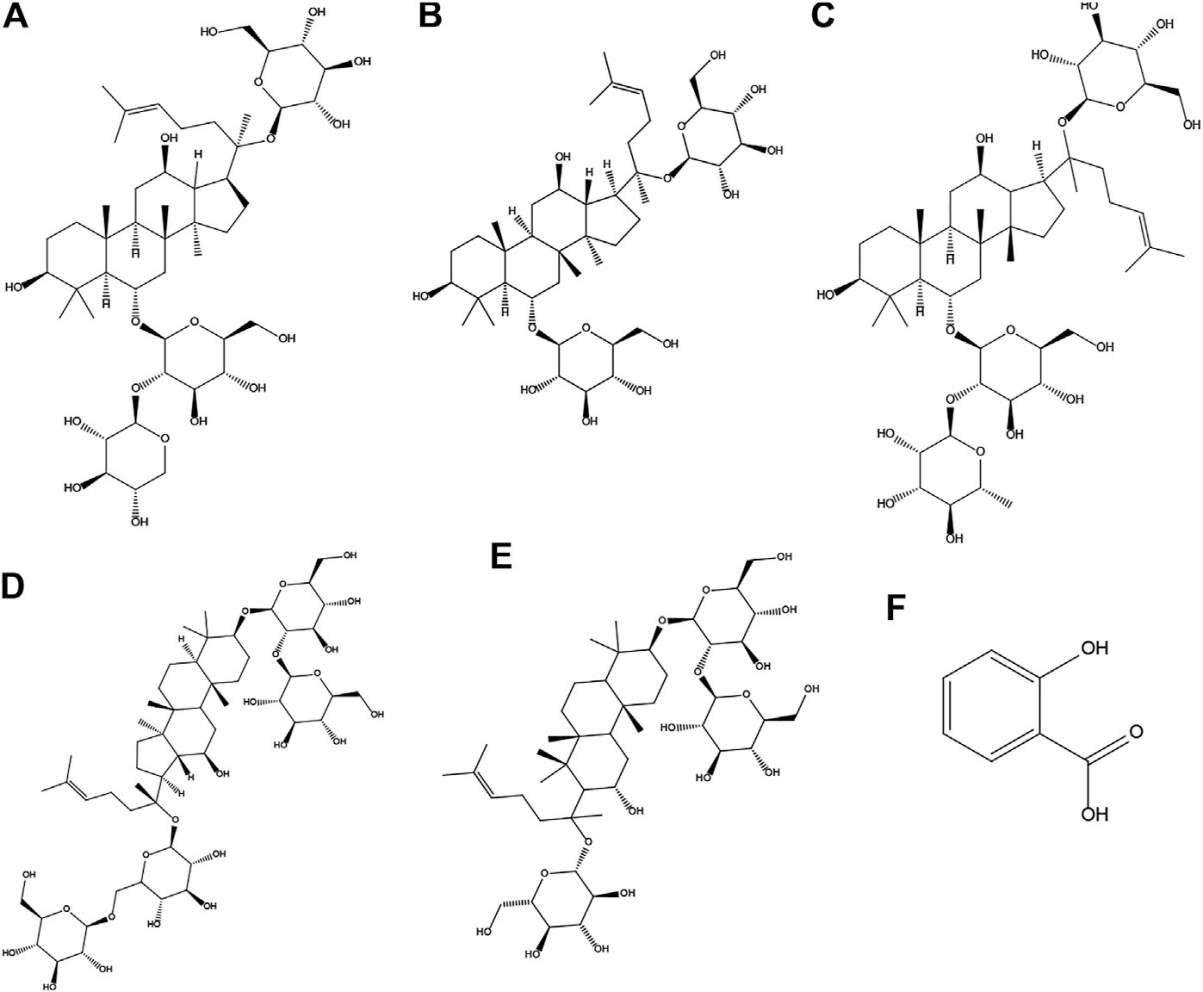
Chemical structures of notoginsenoside R_1_ **(A)** and ginsenoside Rg_1_ **(B)**, Re **(C)**, Rb_1_ **(D)**, Rd **(E),** and aspirin **(F)**.

Panax notoginseng (family Araliaceae) is a well-known and widely used traditional Chinese herb that has been in use for 600 years (Ma et al., 2016; Hu et al., 2019). Panax notoginseng saponins (PNS), containing over 20 types of compounds such as R_1_, Rb_1_, Rg_1_, Re, and Rd (Figure 1), are the major medicinal ingredients extracted from the roots of this herb (Liu et al., 2009; Sun et al., 2018b). In China, some PNS-based drugs have been developed and approved to combat vascular diseases. Aspirin (ASA, Figure 1) is one of the most widely used drugs in the world with approximately 100 billion tablets consumed every year and has been used to prevent myocardial infarction and ischemic stroke (Messerli, 2005). Interestingly, better results in clinic have been obtained when ASA- and PNS-based drugs were taken together. Notably, PNS also exhibited protective effects against gastrointestinal damage caused by ASA (Zhu et al., 2018).

Metabolomics, as a part of system biology, has been extensively applied to profile the endogenous metabolites in organisms and to describe the fluctuations related to the external stimuli or disturbance (Han et al., 2016; Feng et al., 2018; Sun et al., 2018c). Metabolomics can directly reflect the results of genomic or proteinic regulations from a disease and is most closely associated with phenotypic changes (Fang et al., 2017). Metabolomics is sensitive in detecting drug effects because the perturbations of metabolic profiling happened much more easily than the histopathological alterations, which makes it especially suitable for evaluating the integrated regulation effect from herbal products (Xu et al., 2017). Therefore, metabolic profiling has attracted plenty of interest among researchers in the field of herbal research (Du et al., 2015; Zhao et al., 2015; Wang et al., 2019).

The present study aimed to explore the compatibility effects and characteristics of individual and coherent use of PNS and ASA to treat acute blood stasis with the aid of a rat model induced by ice-cold water together with epinephrine injection. The hemorheology profile was applied as an index to investigate the difference between therapeutic effects. GC/MS-based untargeted metabolomics was carried out to describe the inner relationship of pharmacological effects and underlying mechanisms from the perspective of the global metabolic profile to acquire a better understanding of the efficacy differences between combined and single use.

## Materials and Methods

### Materials

ASA enteric capsules were obtained from Tianjin Lisheng Pharmaceutical Co., Ltd (batch number: 1612032, Tianjin, China). PNS was obtained from Yunnan Sanqi Technology Co., Ltd (batch number: 170301, Wenshan, China). PNS contents were determined as notoginsenoside R_1_, 7.4%; ginsenoside Rg_1_, 26.3%; ginsenoside Re, 3.7%; ginsenoside Rb_1_, 27.7%; and ginsenoside Rd, 7.6%. All chemicals were of the highest quality available.

### Experimental Animals and Drug Administration

Male Sprague–Dawley rats (200 ± 10 g) were purchased from Beijing Vital River Laboratory Animal Technology Co. Ltd (license number: SCXK (BJ) 2016–0006). The animals were housed under standard laboratory conditions, and food and tap water were provided ad libitum. The experiment was performed under the United States NIH Guidelines for the Care and Use of Laboratory Animals, and the experimental protocols were approved by the Animal Ethical Committee of Beijing University of Chinese Medicine.

After 7 days of acclimatization, the rats were randomly divided into five groups including the control group, the model group, and three drug-treated groups, which were all administered intragastrically at the scheduled time (9:30–10:30 a.m.) for 10 days. Rats in the control and model groups were supplied with water and food regularly. Rats in the PNS group and the ASA group were given PNS and ASA, respectively. Rats in the PNS combined use with ASA group were coadministered with the above two drugs. The daily doses of PNS or ASA were 31.25 mg/kg and 15.62 mg/kg, respectively (300 mg PNS/60 kg person or 150 mg ASA/60 kg person daily). After the ninth administration, an acute blood stasis rat model was established according to the previously disclosed method with some modifications (Ma et al., 2017) except for the control group. The model rats were subcutaneously injected with adrenaline hydrochloride (0.8 mg/kg). After 2 h, the rats were placed in ice-cold water for 5 min, keeping their heads above the surface, and then reinjected with adrenaline hydrochloride after 2 h from the ice-cold bath. Rats in the control group received an equal volume of saline with subcutaneous injection. Subsequently, the rats were fasted overnight with free access to water and administration continued.

### Sample Preparation and Test Indicators

The rats were anesthetized with chloral hydrate (300 mg/kg) 1 h after the last administration, and blood samples were collected from the abdominal aorta. Blood samples (5 ml) collected into heparin sodium anticoagulant tubes were used to detect the hemorheological indicators. The whole blood viscosity (WBV) was measured with a cone-plate viscometer (SA-9000, Beijing Succeeder Technology Inc., China) at different shear rates maintained at 37°C, and the rest of the blood was centrifuged at 3,000 rpm for 10 min and used for the plasma viscosity (PV) test. Part of the blood sample (2.7 ml) was collected into plastic tubes with 3.8% sodium citrate (citrate/blood: 1/9, v/v) and centrifuged at 1,000 rpm for 10 min to obtain platelet-rich plasma, and platelet-poor plasma was obtained by centrifugation for a further 10 min at 2,500 rpm. The platelets were counted and adjusted to 2.5 × 10^8^ platelets/ml with autologous platelet-poor plasma. The aggregation response was measured according to the turbidimetric method (Schechner et al., 2003). The sodium citrated whole blood (1 ml) was centrifuged at 3,000 rpm for 10 min, and the upper plasma was taken and measured by a platelet aggregation coagulation factor analyzer using the fibrinogen kit (batch No. 20190101, Beijing Succeeder Technology Inc., China) at 37°C.

Additionally, the whole blood samples in K_2_EDTA anticoagulant tubes were immediately centrifuged at 1,000 g for 10 min at 4°C. Aliquots of 200 μL supernatants of plasma samples were separated and centrifuged for 5 min at 4°C and 3,000 g to separate debris or a lipid layer. Each sample aliquot of 50 µL was mixed with 10 µL of internal standard, to which 175 µL of prechilled methanol/chloroform (v/v = 3/1) was added. After the mixture was kept at −20°C in a freezer for 20 min and centrifuged at 14,000 g and 4°C for 20 min, the supernatant was carefully transferred to an auto-sampler vial. All the samples in auto-sampler vials were evaporated briefly to remove chloroform and further lyophilized with a FreeZone freeze dryer. The dried sample was derivatized with 50 µL of methoxyamine (20 mg/ml in pyridine) at 30°C for 2 h, followed by the addition of 50 µL of MSTFA (1% TMCS) containing FAMEs as retention indices and further incubation at 37.5°C for another 1 h. Finally, these samples were used for GC-MS analysis.

### Plasma Metabolomic Profiling and Spectral Acquisition

Plasma metabolomics analysis was performed using an Agilent 7890B gas chromatograph coupled to the GC-MS system (Pegasus HT, Leco Corp., St. Joseph, MO, United States). The separation was carried out on an Rxi-5 MS capillary column (30 m × 250 μm i. d., 0.25-μm film thickness; 5% diphenyl crosslinked 95% dimethylpolysiloxane). A 1-µL derivate was injected with a splitless mode. Helium was used as the carrier gas with a flow rate of 1 ml/min. The oven program was started at 80°C for 2 min, and it was then increased to 300°C at 12°C/min and held for 4.5 min and finally to 320°C at 40°C/min and held for 1 min. The temperatures for injection, the transfer interface, and the ion source were set to 270, 260, and 220°C, respectively. The mass range was set to 50–500 with electron impact ionization (70 eV) and the acquisition rate was 250 spectra per second. The pooled quality control (QC) sample was injected at the beginning of the run to ensure system equilibrium and subsequently every 15 samples to further monitor the stability of the whole analysis.

### Data Processing and Analysis

The raw data generated by GC/MS analysis were processed using XploreMET (v3.0, Metabo-Profile, Shanghai, China) for automated baseline denoizing and smoothing, peak picking and deconvolution, creating a reference database from the pooled QC samples, metabolite signal alignment, missing value correction and imputation, and QC correction. The resulting data matrix containing variables, sample code, and peak area were exported as a csv. file and imported into SIMCA-P software (v13.0, Umetrics, Umea, Sweden) to conduct multivariate statistical analysis. Student’s *t*-test was used for further differentiating variable selection and validation between groups.

### Metabolite Identifications and Pathway Analysis

Metabolite annotation was performed by comparing retention indices and mass spectrum data with the metabolite database JiaLib^™^ which consists of 1,200 mammalian metabolites with a 15-year accumulation. The possible pathways associated with the altered metabolites were obtained from the Kyoto Encyclopedia of Genes and Genomes (KEGG, http://www.genome.jp/kegg/).

### Statistical Analysis

GraphPad Prism 5.0 (GraphPad Software, La Jolla, CA, United States) was used for data analysis. Significance was assessed by the one-way analysis of variance (ANOVA) test, followed by Student’s t-test. *p* values less than 0.05 were considered significant.

## Results

### Influence of Panax Notoginseng Saponins in Combined Use With Aspirin on Acute Blood Stasis in Rats

**FIGURE 2:**
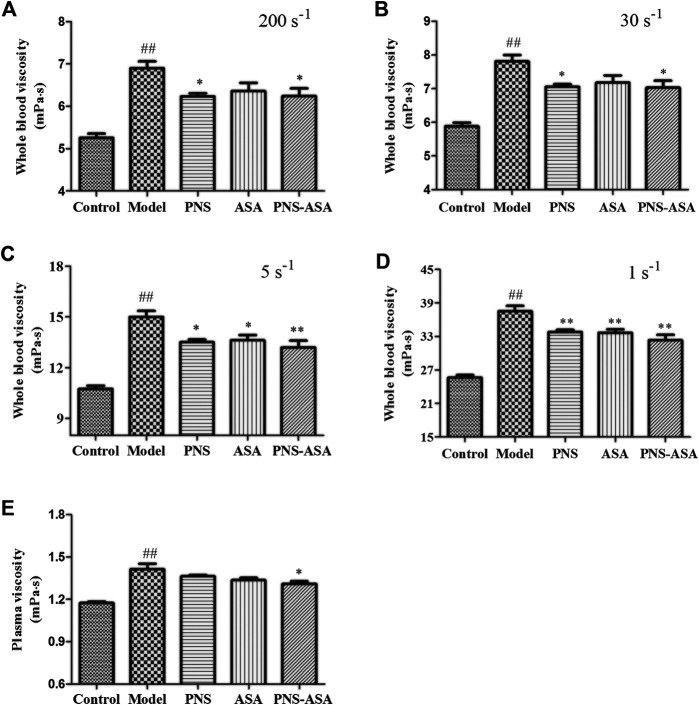
Effects of combined use of PNS and ASA on whole blood viscosity and plasma viscosity in rats with acute blood stasis. **(A–D)** The whole blood viscosity was measured at the shear rates of 200 s^−1^, 30 s^−1^, 5 s^−1^, and 1 s^−1^ in order; **(E)** The plasma viscosity of each tested group. Data were represented as mean ± SD (*n* = 8). *p < 0.05 and **p < 0.01 compared with the model group; ^*##*^ *p* < 0.01 compared with the control group.

To explore the superior efficacy of combined use vs. single use on the hemorheology profile, WBV and PV were selected as indicators in rats with acute blood stasis. Figure 2 shows that WBV at shear rates of 200 s^−1^, 30 s^−1^, 5 s^−1^, and 1 s^−1^ and PV in the model group increased compared to those in the normal control group (*p* < 0.05). However, PNS, ASA, and their combined use could reduce the evaluated WBV at low shear rates (*p* < 0.05). Besides, the PV was decreased after treatment with PNS and ASA in combination (*p* < 0.05).

**FIGURE 3:**
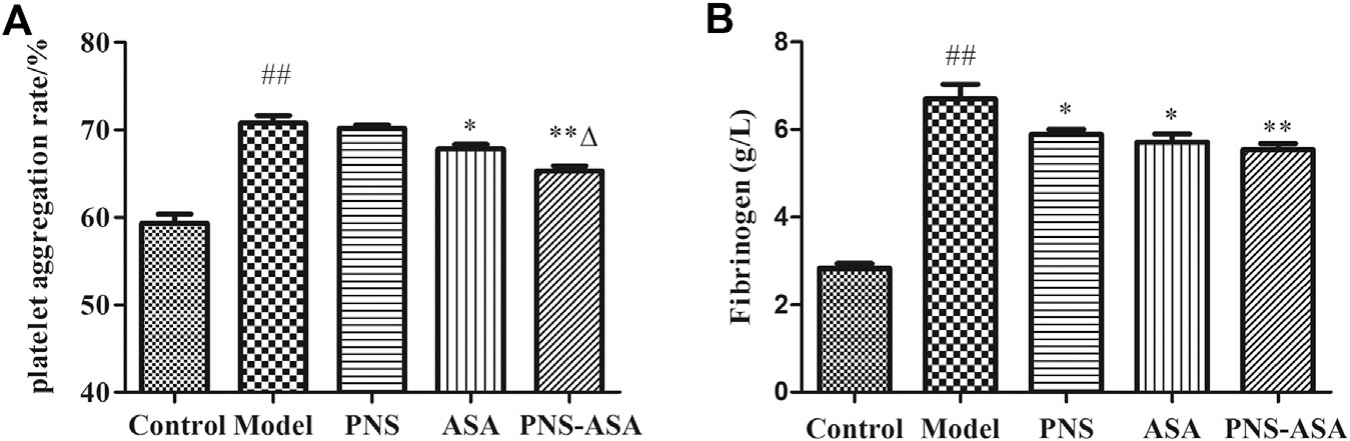
Effects of combined use of PNS and ASA on the rate of platelet aggregation **(A)** and the content of fibrinogen **(B)** in rats with acute blood stasis. Data were represented as mean ± SD (*n* = 8). *p < 0.05 and **p < 0.01 compared with the model group; ^*∆*^ *p* < 0.05 compared with the ASA group; ^*##*^ *p* < 0.01 compared with the control group.

The fibrinogen content and platelet aggregation rate were further detected to explore the improvement of blood rheology. As Figure 3 shows, both ASA and its combined use with PNS decreased platelet aggregation in rats with blood stasis, while the combined use exhibited stronger inhibiting effects than ASA or PNA alone (*p* < 0.05). Also, a significant increase of the plasma fibrinogen level was observed in the model group, and the single and combined use of PNS and ASA decreased the evaluated fibrinogen level in acute blood stasis rats (*p* < 0.05). Taken together, it indicated that the combined use exhibited the superior improvement effects to combat blood stasis vs. single use of PNS or ASA at the same dose.

### GC-TOF/MS Method Validation

The reproducibility and stability of the data were evaluated using QC samples during the whole experimental period. In the multivariate control chart, the position beyond the control limit (±3 standard deviations) is usually indicated as an outlier. The results showed that all points were within the control limit, most of which fluctuates around the *x*-axis within ±2 standard deviations (Supplementary Figure S1). It is presented that all samples conformed to the quality inspection, and the obtained data could be used for further analysis.

### Metabolic Findings in Plasma

The typical GC/MS total ion current (TIC) chromatograms of rat plasma from the control, model, PNS, ASA, and PNS–ASA groups are shown in Supplementary Figure S2, and some significant differences between these five groups were investigated. Based on the JiaLib^™^ database, the majority of the peaks were identified as endogenous metabolites, such as amino acids, carbohydrates, and fatty acids.

**FIGURE 4:**
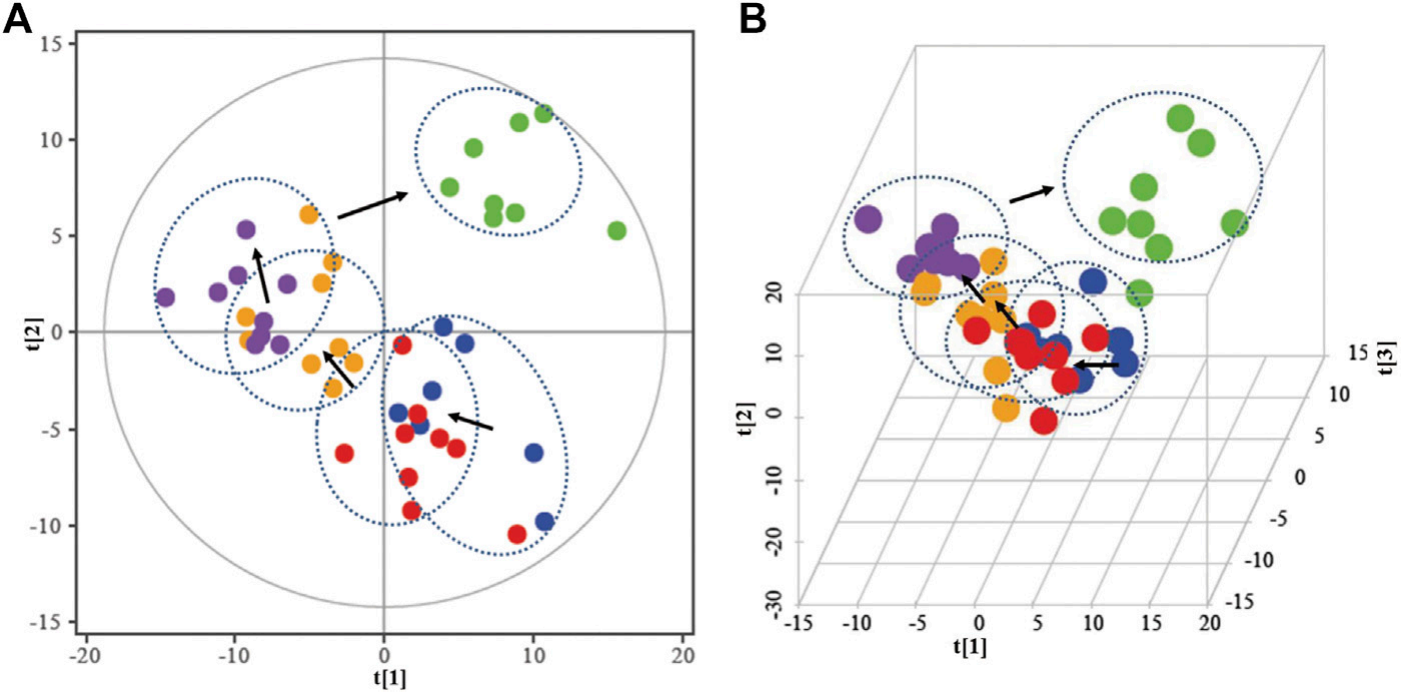
2D-PLS-DA **(A)** and 3D-PLS-DA **(B)** score map in all groups. 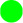
in green indicates in the control group; 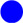
In blue indicates in the model group; 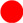
in red indicates in the PNS group; 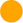
in yellow indicates in the ASA group; 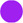
in purple indicates in the combined use group.

To make an overview of the metabolic profile, supervised partial least-squares discriminant analysis (PLS-DA) was employed to perform multiple group comparisons. In Figure 4, the separation trend was observed in a PLS-DA score plot. The relative distance of single and combined use of PNS and ASA was separated from the model group with a trend closer to the control group with the score as an index, suggesting that drug intervention recovered the abnormal metabolism in the blood stasis rats to the normal state. Notably, the relative distance of the combined use was closer to the normal group than the single-use group, indicating better regulatory effects of combined application to treat blood stasis.

### Potential Discriminatory Metabolites Selection

Pattern recognition was applied to analyze the plasma metabolites to capture subtle metabolic perturbations in rats. Orthogonal partial least squares discriminant analysis (OPLS-DA) was used to establish a model to discriminate two predefined groups. The normalized data set of the characteristic metabolites was used as input data, with high cumulative *R* ^2^ and Q^2^ values, and a permutation test (1,000 iterations) was used to avert the overfitting of the model (Supplementary Figure S3).

**FIGURE 5:**
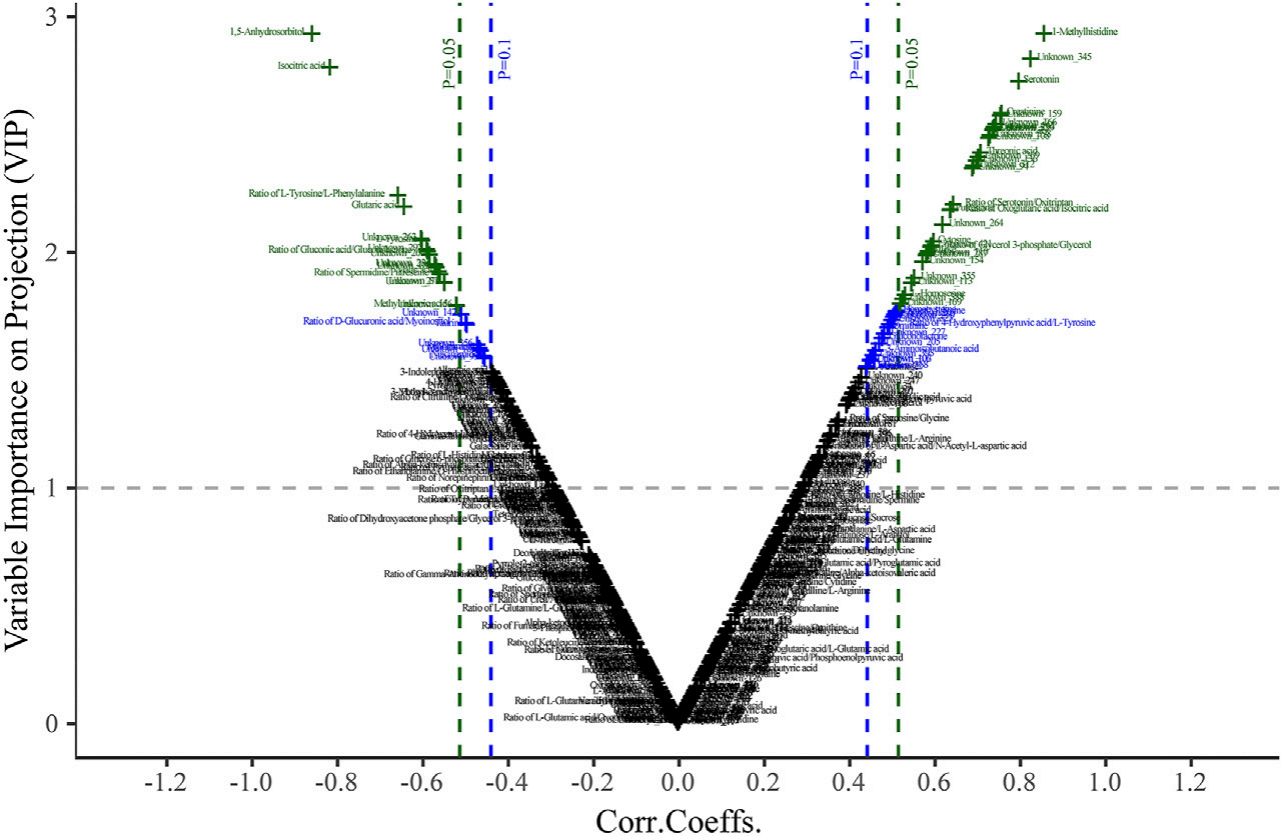
Plasma volcano-plot between the control and model groups. The x- and *y*-axis represent correlation coefficients (Corri.Coeffs.) and variable importance on projection (VIP) in order.

The discriminatory metabolites that contributed to the separation between the normal control and model groups were selected based on the variable importance in projection (VIP) threshold and *p*-value. Variables with VIP >1.0 were highlighted as the optional biomarkers, and these variables were further filtered by Student’s *t*-test to select the features with a significant difference (*p* < 0.05) between the two groups and labeled in the volcano-plot (Figure 5). The combined results revealed that there were twelve highlighted metabolites in total that could be kept as the potential biomarkers including five amino acids, two carbohydrates and organic acids, and one alkyl amine, indole, and fatty acid (Table 1).

**TABLE 1:** Potential discriminatory metabolites of plasma in the rats with acute blood stasis.

No.	Metabolite	HMDBID	Class	VIP^a^	FC^b^	*p* value			
						Model	PNS/model	ASA/model	PNS + ASA/model
1	Putrescine	01414	Alkylamines	2.2	1.270	↑	—	↓	↓
2	1-Methylhistidine	00001	Amino acids	2.9	2.083	↑	—	—	—
3	Creatinine	00562	Amino acids	2.6	2.188	↑	↓	↓	↓
4	Tyrosine	00158	Amino acids	2.0	0.750	↓	↑	↑	↑
5	Homoserine	00719	Amino acids	1.8	1.347	↑	—	—	↓
6	Homocysteine	00742	Amino acids	1.7	1.439	↑	—	—	↓
7	1,5-Anhydrosorbitol	02712	Carbohydrates	2.9	0.264	↓	—	↑	↑
8	Threonic acid	00943	Carbohydrates	2.4	1.831	↑	↓	↓	↓
9	Arachidonic acid	01043	Fatty acids	1.1	1.392	↑	↓	↓	↓
10	Serotonin	00259	Indoles	2.7	2.433	↑	↓	↓	↓
11	Isocitric acid	00193	Organic acids	2.7	0.461	↓	—	—	—
12	Glutaric acid	00661	Organic acids	2.2	0.738	↓	—	—	—

a VIP (variable importance in projection) was obtained from the OPLS-DA model between the model and control groups.

b FC (fold change) was calculated as the ratio of metabolite level between the model and control groups. FC with a value >1.0 indicated a relatively higher level while a value <1.0 indicated a relatively lower level present in the model group than the control group. The symbol “—” denotes that the metabolite did not significantly change.

Additionally, it was found that single use of PNS and ASA reversed five and seven abnormally altered endogenous metabolite levels, respectively. However, the combined use reversed nine endogenous metabolite levels. The drugs reduced the abnormal changes of these endogenous metabolites of plasma with the tendency to the normal levels, thereby exerting the effect to combat blood stasis. The fact that the combined use regulated the highest amount of endogenous metabolites indicated that the combined use had better treatment efficacy than single use.

### Metabolic Pathway Analysis and the Influence of Drug Treatments

**FIGURE 6:**
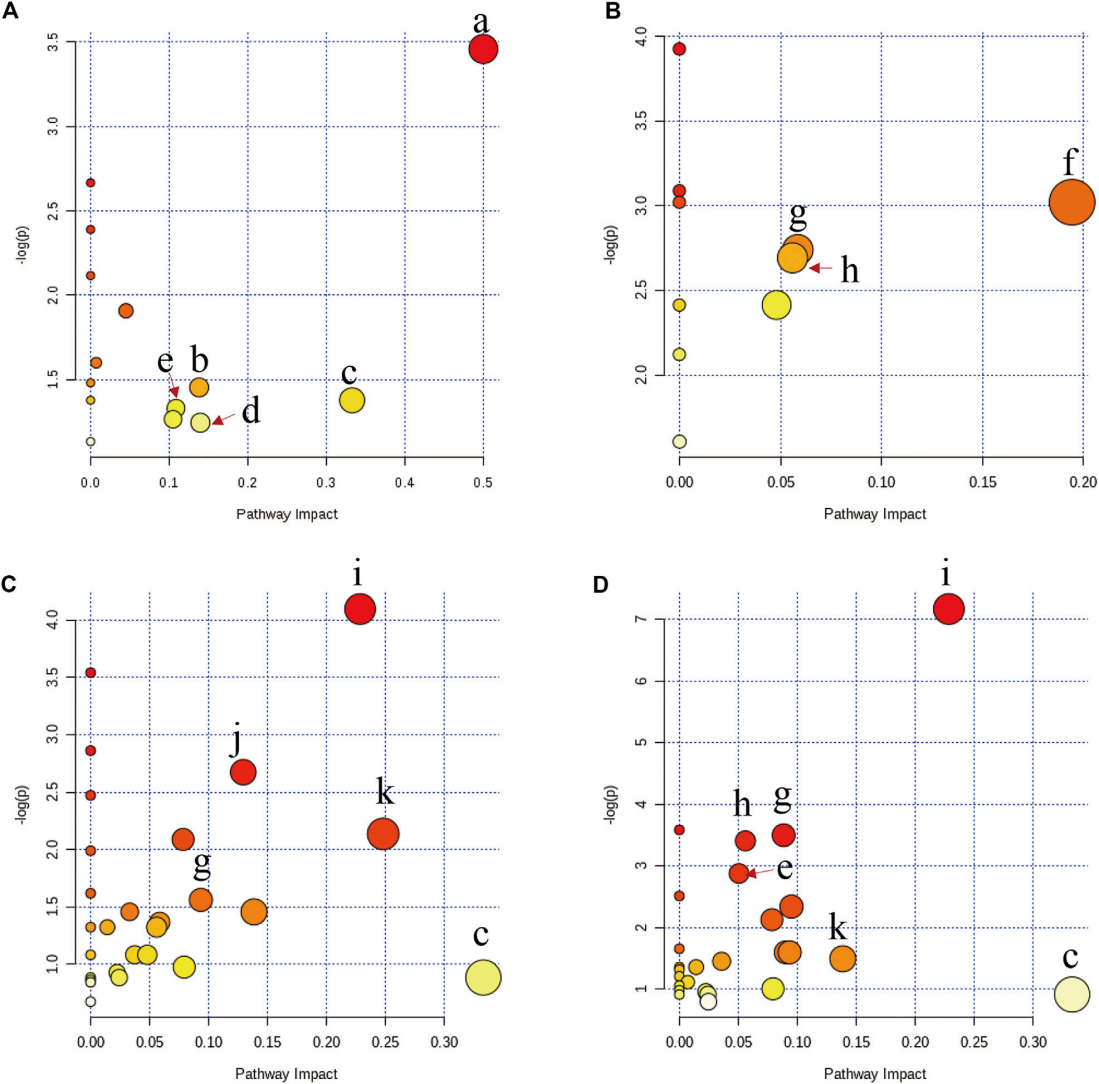
Altered metabolic pathways in rats with acute blood stasis **(A)** and the influence of disturbed metabolic pathways by PNS treatment **(B)**, ASA treatment **(C),** and the treatment of their combined use **(D)** by bubble plot. The characters of a to k represent the phenylalanine, tyrosine, and tryptophan biosynthesis, cysteine and methionine metabolism, arachidonic acid metabolism, tyrosine metabolism, arginine and proline metabolism, nicotinate and nicotinamide metabolism, TCA cycle, β-alanine metabolism, arginine biosynthesis, and inositol phosphate, starch, and sucrose metabolism, respectively.

The discriminatory metabolites between the model group and the other groups were imported into MetaboAnalyst 4.0 (https://www.metaboanalyst.ca/) to perform pathway analysis. In Figure 6A, the metabolic disorders in rats with acute blood stasis were related to the biosynthesis or metabolism pathway including phenylalanine, tyrosine, and tryptophan biosynthesis, cysteine and methionine metabolism, and arachidonic acid metabolism, suggesting that acute blood stasis might be a vascular disease with a complex pathogenesis involving multiple metabolic pathways. PNS affected metabolic pathways involving nicotinate and nicotinamide metabolism, TCA cycle, and β-Alanine metabolism (Figure 6B). ASA affected metabolic pathways involving β-Alanine metabolism, arginine biosynthesis, and inositol phosphate, starch, and sucrose metabolism (Figure 6C). The combined use of both drugs affected metabolic pathways involving cysteine and methionine metabolism, arachidonic acid metabolism, tyrosine metabolism, β-Alanine metabolism, arginine biosynthesis, and inositol phosphate, starch, and sucrose metabolism (Figure 6D).

## Discussion

The acute blood stasis rat model is usually induced by the ice-cold water together with epinephrine injections and can mimic the patients’ prethrombotic state to some extent and contribute greatly to the investigation of the underlying mechanisms of blood stasis (Li et al., 2009; Zou et al., 2015). Hemorheology could generally reflect the changes in blood flow, stagnation, and viscosity from the alternation of the tangible or intangible components. Blood stagnation will bring the deterioration of blood fluidity which directly affects the supply of blood to the organs and further induce vascular diseases (Li et al., 2009). Blood viscosity is mainly related to the change in fibrinogen and results in the rise of PV and WBV (Tsuda et al., 1996; Stanger 2005; Zheng et al., 2014). Here, the results of the conventional pharmacological assessment showed that the combined use of PNS and ASA exhibited a better effect while combating blood stasis than PNS or ASA alone at the same dose. Thus, we speculated that their combined use might reduce the dosage of ASA in clinical practice.

To provide an insight into the superior effects of the combined use of PNS and ASA on acute blood stasis, an untargeted metabolomics approach was conducted to further explore the cooperative protection mechanism from the overall metabolic level. Compared with blood stasis rats, the metabolic network produced significant changes after treatment with PNS and ASA separately and coherently. Furthermore, combined use regulated more identified potential biomarkers than single use in this study, which supported our results of blood rheology. Moreover, this finding also supported our previous results of the pharmacokinetics and cell experiment (Tian et al., 2017).

Amino acids play a critical role in human metabolism and are widely used as substrates for many metabolic pathways including the tricarboxylic acid (TCA) cycle, protein synthesis, and metabolism regulators. Among these metabolites, tyrosine is one of the essential amino acids which could be converted from phenylalanine and is related to phenylalanine, tyrosine, and tryptophan biosynthesis. Consistent with a recent report showing that the tyrosine level decreased in rats with blood stasis (Ma et al., 2017), we concluded that the pathological state leads to the unsuccessful conversion of phenylalanine into tyrosine and evaluated the ratio of phenylalanine/tyrosine. Thus, the altered levels of tyrosine could be used as an important index of disturbance of amino acid metabolism induced by blood stasis. In this study, PNS and/or ASA reversed the reduced tyrosine level in rats with blood stasis, indicating that they could improve this metabolism disorder of blood stasis.

Homocysteine is an intermediary amino acid formed during the metabolism of methionine to cysteine and plays a key role in the transsulfuration pathway (Mcmenamin et al., 2009; Rafii et al., 2009). Many studies showed that homocysteine metabolism disorder is accompanied by several adverse clinical outcomes, such as the increased risk of thrombotic vascular diseases (Ganguly and Alam, 2015; Martí-Carvajal et al., 2015; Chen et al., 2016). The elevated homocysteine could also lead to an increase in tissue S-adenosylhomocysteine concentration, a feedback inhibitor of all methyltransferase reactions, including those involved in DNA, protein, and lipid methylation. Homocysteine clearance is critical to avoid this inhibition (Finkelstein, 2007). In this study, the combined use of PNS and ASA exhibited a good regulatory effect for the plasma homocysteine level, whereas no such effect was observed in PNS or ASA individually. The result, furthermore, supported the fact that the protective effect of combined use on vascular diseases is better than that of single use.

As another important amino acid, creatinine is usually produced at a fairly constant rate in the body. The presence of creatinine in blood plasma is indicative of tissue damage, and its level is widely used to evaluate renal function (Li et al., 2007; Zou et al., 2015; Yoshimi et al., 2016). The kidney develops functional disorders owing to the relative lack of blood supply as the blood stasis or thrombosis occurs (Haase et al., 2009). As a result, nitrogenous wastes including creatinine cannot be excreted from the kidney. In the model group, increased creatinine indicated that renal injury had occurred, which was in accordance with a previous experimental result (Yan et al., 2009). Both PNS and ASA could reverse the abnormally elevated creatinine level. The present finding also confirmed that PNS and ASA improved the hemodynamics to some extent, thereby enhancing the blood supply for the kidney to help restore its function.

Putrescine is a well-known biogenic amine involved in the arginine and proline metabolic pathways (Di Franco et al., 2018). Arginine can produce ornithine and urea *via* arginolytic enzymes. The metabolic pathway is associated with creatinine metabolism and the urea cycle. Putrescine is an intermediate metabolite of the urea cycle. Fumarate is formed in the urea cycle, thus linking the urea cycle to the TCA cycle which involves the glucose aerobic oxidation and the major pathways of fat and amino acid metabolisms (Zou et al., 2015; Lundgren et al., 2018). Our results suggested that energy production during aerobic respiration was suppressed in rats with blood stasis. However, ASA or its combined use with PNS could reverse the abnormally elevated level of putrescine. We boldly speculated that the disturbed metabolic rate of energy metabolism was restored until it was close to the normal level.

Carbohydrates are also an important class of metabolites related to living substance and energy metabolism. Threonic acid is a breakdown product of additive ascorbic acid, which plays several important cellular and biochemical roles as an antioxidant owing to its high reducing potential (Simon et al., 1998). In a previous study, oxidative stress was found to be an important pathogenesis of blood stasis (Zou et al., 2015). PNS could help to restrain the oxidative stress (Zhang et al., 2019). We observed that the level of threonic acid had been significantly increased in the acute blood stasis models. So, we speculated that ascorbic acid might be converted to threonic acid, resulting in a great reduction in the content. Furthermore, 1,5-anhydrosorbitol could be associated with the TCA cycle. Here, PNS combined with ASA exhibiting the callback regulations of these two metabolites might be involved with their antioxidant stress and energy metabolism.

Arachidonic acid is released from the membrane *via* the cleavage by phospholipase A2 in response to stimuli (Burstein et al., 1982; Liu et al., 2019). Thromboxane A2, a product of arachidonic acid with the aid of cyclooxygenase, is involved in vascular contraction and has been implicated in platelet activation (Rosenfeld et al., 2010). It has been reported that platelets binding to a blood clot will lead to the release of serotonin, which is an important vasoconstrictor and regulates hemostasis and clotting (Ilveskero and Lassila, 2003). Our findings showed that PNS combined with ASA minimized the abnormal increase of arachidonic acid and serotonin compared to single use, indicating that the combined use exhibited better therapeutic effects on preventing thrombosis.

**FIGURE 7:**
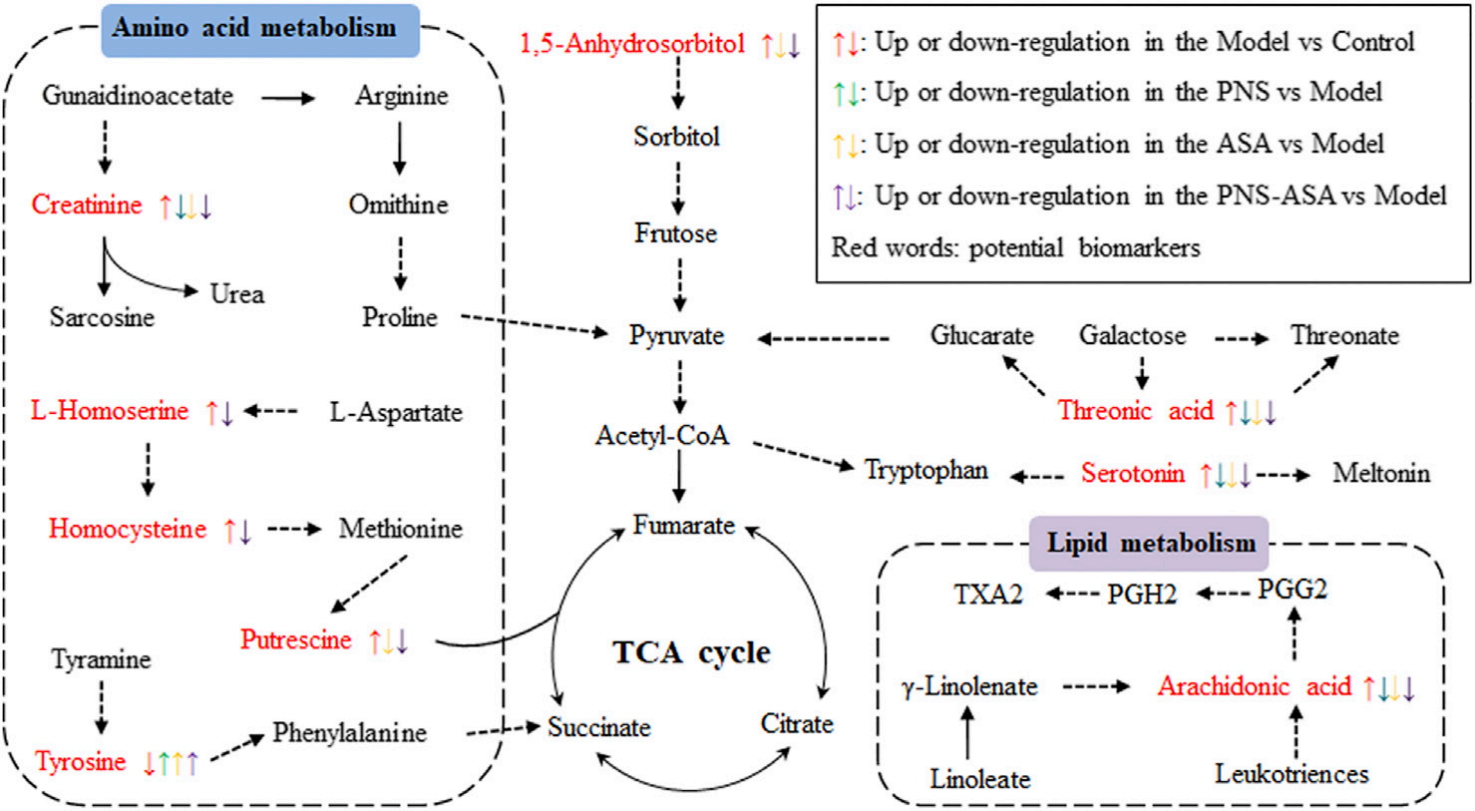
Potential metabolic pathways affected by acute blood stasis and the influence on the treatments of PNS, ASA, and the combined use of both drugs. Red words indicate potential biomarkers of acute blood stasis.

Taken together, our data demonstrated that PNS combined with ASA exhibited better improvement on the anti–blood stasis effect than PNS or ASA alone. The main metabolic pathways included were proposed as follows: amino acid metabolism, lipid metabolism, energy metabolism, etc (Figure 7). We are currently endeavoring to quantitatively analyze these possible biomarkers associated with blood stasis on these metabolic pathways to further confirm that the combined use exhibits a stronger regulatory effect than single use.

## Conclusion

The present work demonstrated that PNS could cooperate with ASA to improve blood rheology in the status of acute blood stasis. Untargeted metabolomics revealed that there were significant differences in the metabolic network between rats with blood stasis and rats after being treated with PNS, ASA, and their combined application. We further speculated that the superior efficacy of PNS in combined use with ASA was mainly related to amino acid metabolism, lipid metabolism, and energy metabolism. Our results also suggest that the combined use of PNS and ASA should pay attention to the dosage of ASA in clinical practice.

## Data Availability

The original contributions presented in the study are included in the article/Supplementary Material; further inquiries can be directed to the corresponding author.

## References

[B1] Borrelli F. Izzo A. A. (2009). Herb-drug interactions with St. John's wort (*Hypericum perforatum*): an update on clinical observations. AAPS J. 11, 710–727. 10.1208/s12248-009-9146-8 19859815 PMC2782080

[B2] Brantley S. J. Argikar A. A. Lin Y. S. Nagar S. Paine M. F. (2014). Herb-drug interactions: challenges and opportunities for improved predictions. Drug Metab. Dispos. 42, 301–317. 10.1124/dmd.113.055236 24335390 PMC3935140

[B3] Burstein S. Hunter S. A. Sedor C. Shulman S. (1982). Prostaglandins and cannabis-IX. Biochem. Pharmacol. 31, 2361–2365. 10.1016/0006-2952(82)90530-5 6289843

[B4] Chen H. Chen X. Hong X. Liu C. Huang H. Wang Q. (2016). Maternal exposure to ambient PM2.5 exaggerates fetal cardiovascular maldevelopment induced by homocysteine in rats. Environ. Toxicol. 32, 877–889. 10.1002/tox.22287 27203204

[B5] Di Franco M. Lucchino B. Conti F. Valesini G. Spinelli F. R. (2018). Asymmetric dimethyl arginine as a biomarker of atherosclerosis in rheumatoid arthritis. Mediators Inflamm. 2018, 3897295. 10.1155/2018/3897295 29576746 PMC5822828

[B6] Du L. N. Xie T. Xu J. Y. Kang A. Di L. Q. Shan J. J. (2015). A metabolomics approach to studying the effects of Jinxin oral liquid on RSV-infected mice using UPLC/LTQ-Orbitrap mass spectrometry. J. Ethnopharmacol. 174, 25–36. 10.1016/j.jep.2015.07.040 26234176

[B7] Fang L. Gu C. Liu X. Xie J. Hou Z. Tian M. (2017). Metabolomics study on primary dysmenorrhea patients during the luteal regression stage based on ultra performance liquid chromatography coupled with quadrupole-time-of-flight mass spectrometry. Mol. Med. Rep. 15, 1043–1050. 10.3892/mmr.2017.6116 28098892 PMC5367332

[B8] Fasinu P. S. Bouic P. J. Rosenkranz B. (2012). An overview of the evidence and mechanisms of herb-drug interactions. Front. Pharmacol. 3, 1–19. 10.3389/fphar.2012.00069 22557968 PMC3339338

[B9] Feng X. Li M. H. Xia J. Deng Ba D. J. Ruan L. Y. Xing Y. X. (2018). Tibetan medical formula shi-wei-gan-ning-pill protects against carbon tetrachloride-induced liver fibrosis—an NMR-based metabolic profiling. Front. Pharmacol. 9, 965. 10.3389/fphar.2018.00965 30210344 PMC6123542

[B10] Finkelstein J. D. (2007). Metabolic regulatory properties of S-adenosylmethionine and S-adenosylhomocysteine. Clin. Chem. Lab. Med. 45, 1694–1699. 10.1515/cclm.2007.341 17963455

[B11] Ganguly P. Alam S. F. (2015). Role of homocysteine in the development of cardiovascular disease. Nutr. J. 14, 1–65. 10.1186/1475-2891-14-6 25577237 PMC4326479

[B12] Haase M. Haase-Fielitz A. Bellomo R. Devarajan P. Story D. Matalanis G. (2009). Sodium bicarbonate to prevent increases in serum creatinine after cardiac surgery: a pilot double-blind, randomized controlled trial*. Crit. Care Med. 37, 39–47. 10.1097/ccm.0b013e318193216f 19112278

[B13] Han Y. Li Y. Wang Y. Gao J. Xia L. Hong Y. (2016). Comparison of fresh, dried and stir-frying gingers in decoction with blood stasis syndrome in rats based on a GC-TOF/MS metabolomics approach. J. Pharm. Biomed. Anal. 129, 339–349. 10.1016/j.jpba.2016.07.021 27454085

[B14] Hu S. Liu T. Wu Y. Yang W. Hu S. Sun Z. (2019). Panax notoginseng saponins suppress lipopolysaccharide‐induced barrier disruption and monocyte adhesion on bEnd.3 cells *via* the opposite modulation of Nrf2 antioxidant and NF‐κB inflammatory pathways. Phytother. Res. 33, 3163–3176. 10.1002/ptr.6488 31468630

[B15] Ilveskero S. Lassila R. (2003). Abciximab inhibits procoagulant activity but not the release reaction upon collagen- or clot-adherent platelets. J. Thromb. Haemost. 1, 805–813. 10.1046/j.1538-7836.2003.00136.x 12871419

[B16] Li H. X. Han S. Y. Wang X. W. Ma X. Zhang K. Wang L. (2009). Effect of the carthamins yellow from carthamus tinctorius L. on hemorheological disorders of blood stasis in rats. Food Chem. Toxicol. 47, 1797–1802. 10.1016/j.phymed.2015.01.006 19406191

[B17] Li L. Wang J. Ren J. Xiang J. Tang Y. Liu J. (2007). Metabonomics analysis of the urine of rats with Qi deficiency and blood stasis syndrome based on NMR techniques. Chin. Sci. Bull. 52, 3068–3073. 10.1007/s11434-007-0389-4

[B18] Liu H. Yang J. Du F. Gao X. Ma X. Huang Y. (2009). Absorption and disposition of ginsenosides after oral administration of Panax notoginseng extract to rats. Drug Metab. Dispos. 37, 2290–2298. 10.1124/dmd.109.029819 19786509

[B19] Liu R. Chen Y. Fu W. Wang S. Cui Y. Zhao X. (2019). Fexofenadine inhibits TNF signaling through targeting to cytosolic phospholipase A2 and is therapeutic against inflammatory arthritis. Ann. Rheum. Dis. 78, 1524–1535. 10.1136/annrheumdis-2019-215543 31302596 PMC8157820

[B20] Lundgren J. Sandqvist A. Hedeland M. Bondesson U. Wikström G. Rådegran G. (2018). Alterations in plasma L-arginine and methylarginines in heart failure and after heart transplantation. Scand. Cardiovasc. J. 52, 196–204. 10.1080/14017431.2018.1459823 29648475

[B21] Ma J. Mi Y. Li Q. Chen L. Du L. He L. (2016). Reduction, methylation, and translocation of arsenic in Panax notoginseng grown under field conditions in arsenic-contaminated soils. Sci. Total Environ. 550, 893–899. 10.1016/j.scitotenv.2016.01.188 26851761

[B22] Ma Q. Li P. L. Hua Y. L. Ji P. Yao W. L. Zhang X. S. (2017). Effects of Tao-Hong-Si-Wu decoction on acute blood stasis in rats based on a LC-Q/TOF-MS metabolomics and network approach. Biomed. Chromatogr. 32, e4144. 10.1002/bmc.4144 29149492

[B23] Martí-Carvajal A. J. Solà I. Lathyris D. Salanti G. (2015). Homocysteine lowering interventions for preventing cardiovascular events. Cochrane DB. Syst. Rev. 1, CD006612. 10.1002/14651858.CD006612.pub2 25590290

[B24] Mcmenamin M. E. Himmelfarb J. Nolin T. D. (2009). Simultaneous analysis of multiple aminothiols in human plasma by high performance liquid chromatography with fluorescence detection. J. Chromatogr. B 877, 3274–3281. 10.1016/j.jchromb.2009.05.046 19515618

[B25] Messerli F. H. (2005). Aspirin: a novel antihypertensive drug? J. Am. Coll. Cardiol. 46, 984–985. 10.1016/j.jacc.2005.06.050 16168279

[B26] Murray J. Picking D. Lamm A. McKenzie J. Hartley S. Watson C. (2016). Significant inhibitory impact of dibenzyl trisulfide and extracts of Petiveria alliacea on the activities of major drug-metabolizing enzymes *in vitro*: an assessment of the potential for medicinal plant-drug interactions. Fitoterapia 111, 138–146. 10.1016/j.fitote.2016.04.011 27105957

[B27] Rafii M. Elango R. House J. D. Courtney-Martin G. Darling P. Fisher L. (2009). Measurement of homocysteine and related metabolites in human plasma and urine by liquid chromatography electrospray tandem mass spectrometry. J. Chromatogr. B. 877, 3282–3291. 10.1016/j.jchromb.2009.05.002 19481985

[B28] Rosenfeld L. Grover G. J. Stier C. T. Jr (2010). Ifetroban sodium: an effective TxA2/PGH2 receptor antagonist. Cardiovasc. Ther. 19, 97–115. 10.1111/j.1527-3466.2001.tb00058.x 11484065

[B29] Schechner V. Shapira I. Berliner S. Comaneshter D. Hershcovici T. Orlin J. (2003). Significant dominance of fibrinogen over immunoglobulins, C-reactive protein, cholesterol and triglycerides in maintaining increased red blood cell adhesiveness/aggregation in the peripheral venous blood: a model in hypercholesterolaemic patients. Eur. J. Clin. Invest*.* 33, 955–961. 10.1046/j.1365-2362.2003.01260.x 14636298

[B30] Simon J. A. Hudes E. S. Browner W. S. (1998). Serum ascorbic acid and cardiovascular disease prevalence in U.S. adults. Epidemiology 9, 316–321. 10.1097/00001648-199805000-00017 9583425

[B31] Stanger O. (2005). Editorial [hot topic: clinical investigations (endothelial function and thrombosis), intracellular metabolism and cell culture studies with infectious agents (guest editor: Olaf Stanger)]. Current Drug Meta. 6, 1. 10.2174/1389200052997375

[B32] Sun Z. Wu Y. Liu S. Hu S. Zhao B. Li P. (2018a). Effects of Panax notoginseng saponins on esterases responsible for aspirin hydrolysis *in vitro* . Int. J. Mol. Sci. 19, 3144. 10.3390/ijms19103144 PMC6213075 30322078

[B34] Sun Z. Wu Y. Yang B. Zhu B. Hu S. Lu Y. (2018b). Inhibitory influence of Panax notoginseng Saponins on aspirin hydrolysis in human intestinal caco-2 cells. Molecules 23, 455. 10.3390/molecules23020455 PMC6016969 29463025

[B33] Sun J. Jing S. Jiang R. Wang C. Zhang C. Chen J. (2018c). Metabolomics study of the therapeutic mechanism of schisandra chinensis lignans on aging rats induced by D-galactose. Clin. Interv. Aging 13, 829–841. 10.2147/cia.s163275 29750025 PMC5935080

[B35] Tian Z. Pang H. Du S. Lu Y. Zhang L. Wu H. (2017). Effect of panax notoginseng saponins on the pharmacokinetics of aspirin in rats. J. Chromatogr. B 1040, 136–143. 10.1016/j.jchromb.2016.12.007 27978468

[B36] Tsuda Y. Satoh K. Kitadai M. Takahashi T. Izumi Y. Hosomi N. (1996). Effects of pravastatin sodium and simvastatin on plasma fibrinogen level and blood rheology in type II hyperlipoproteinemia, Atherosclerosis 122, 225–233. 10.1016/0021-9150(95)05757-9 8769685

[B37] Wang J. Xiong X. Feng B. (2014). Aspirin resistance and promoting blood circulation and removing blood stasis: current situation and prospectives. Evidence-Based Complement. Altern. Med. 2014, 1–11. 10.1155/2014/954863 PMC3948594 24696702

[B38] Wang Y. Zhao H. Liu Y. Guo W. Bao Y. Zhang M. (2019). GC-MS-based metabolomics to reveal the protective effect of gross saponins of Tribulus terrestris fruit against ischemic stroke in rat. Molecules 24, 793. 10.3390/molecules24040793 PMC6412276 30813246

[B39] Xiong X. (2015). Integrating traditional Chinese medicine into western cardiovascular medicine: an evidence-based approach. Nat. Rev. Cardiol. 12, 374. 10.1038/nrcardio.2014.177-c1 25917150

[B40] Xu J. J. Zheng X, J. Cheng K. Chang X. R. Shen G. P. Liu M. (2017). NMR-based metabolomics reveals alterations of electro-acupuncture stimulations on chronic atrophic gastritis rats. Sci. Rep. 7, 45580. 10.1038/srep45580 28358020 PMC5372362

[B41] Yan B. Ji-Ye A. Wang G. Zhu X. Zha W. (2009). Metabonomic phenotype and identification of “heart blood stasis obstruction pattern” and “qi and yin deficiency pattern” of myocardial ischemia rat models. Sci. China Ser. C. 52, 1081–1090. 10.1007/s11427-009-0136-y 19937207

[B42] Yoshimi N. Futamura T. Kakumoto K. Salehi A. M. Sellgren C. M. Holmén-Larsson J. (2016). Blood metabolomics analysis identifies abnormalities in the citric acid cycle, urea cycle, and amino acid metabolism in bipolar disorder. BBA. Clin. 5, 151–158. 10.1016/j.bbacli.2016.03.008 27114925 PMC4832124

[B43] Zhang M. Guan Y. Xu J. Qin J. Li C. Ma X. (2019). Evaluating the protective mechanism of panax notoginseng saponins against oxidative stress damage by quantifying the biomechanical properties of single cell. Analytica Chim. Acta 1048, 186–193. 10.1016/j.aca.2018.10.030 30598149

[B44] Zhao T. Zhang H. Zhang X. Zhao T. Lan H. Y. Liang Q. (2015). Metabolomic and lipidomic study of the protective effect of Chaihuang-Yishen formula on rats with diabetic nephropathy. J. Ethnopharmacol. 166, 31–41. 10.1016/j.jep.2015.02.019 25698246

[B45] Zheng C. Z. Jia M. Zhang L. B. Yao W. F. Su S. L. Tang Y. P. (2014). Comparison on activating blood circulation effects of Jiaoai Decoction and Siwu Decoction on acute blood stasis rats. Chin. Trad. Herb. Drugs 45, 2652–2657. 10.7501/j.issn.0253-2670.2014.18.015

[B46] Zhu B. Zhang W. Lu Y. Hu S. Gao R. Sun Z. (2018). Network pharmacology-based identification of protective mechanism of Panax notoginseng saponins on aspirin induced gastrointestinal injury. Biomed. Pharmacother. 105, 159–166. 10.1016/j.biopha.2018.04.054 29857294

[B47] Zou Z. J. Liu Z. H. Gong M. J. Han B. Wang S. M. Liang S. W. (2015). Intervention effects of puerarin on blood stasis in rats revealed by a 1H NMR-based metabonomic approach. Phytomedicine 22, 333–343. 10.1016/j.phymed.2015.01.006 25837270

