# Supplementary figures and images for: Comparative Analysis of Compatibility Influence on Invigorating Blood Circulation for Combined Use of Panax Notoginseng Saponins and Aspirin Using Metabolomics Approach
Source: Front Pharmacol. 2021 Apr 30;12:544002. doi: 10.3389/fphar.2021.544002 (PMC8120290; doi:10.3389/fphar.2021.544002)

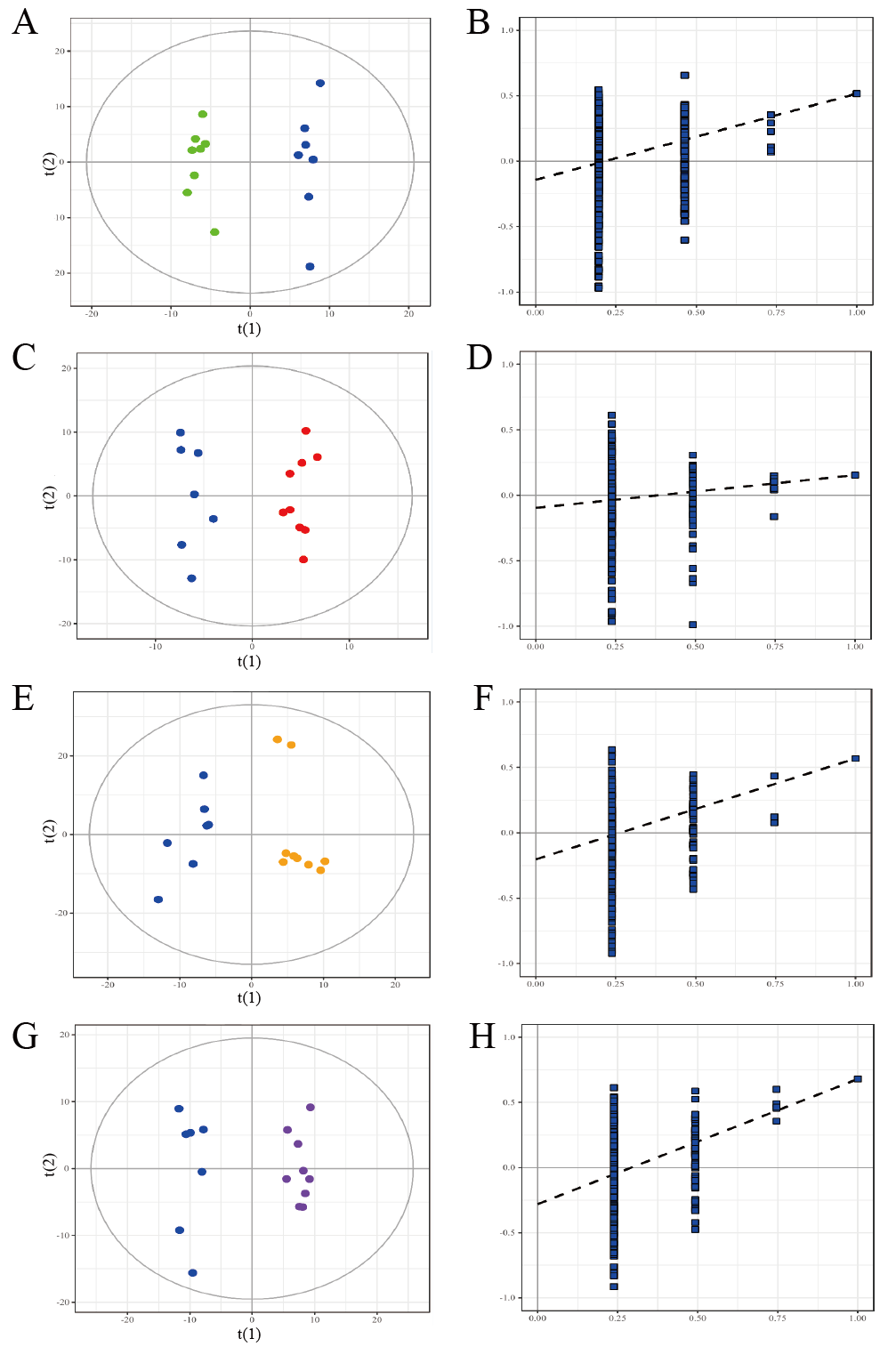

Supplement: Supplementary file 1 [file Image3.TIF]

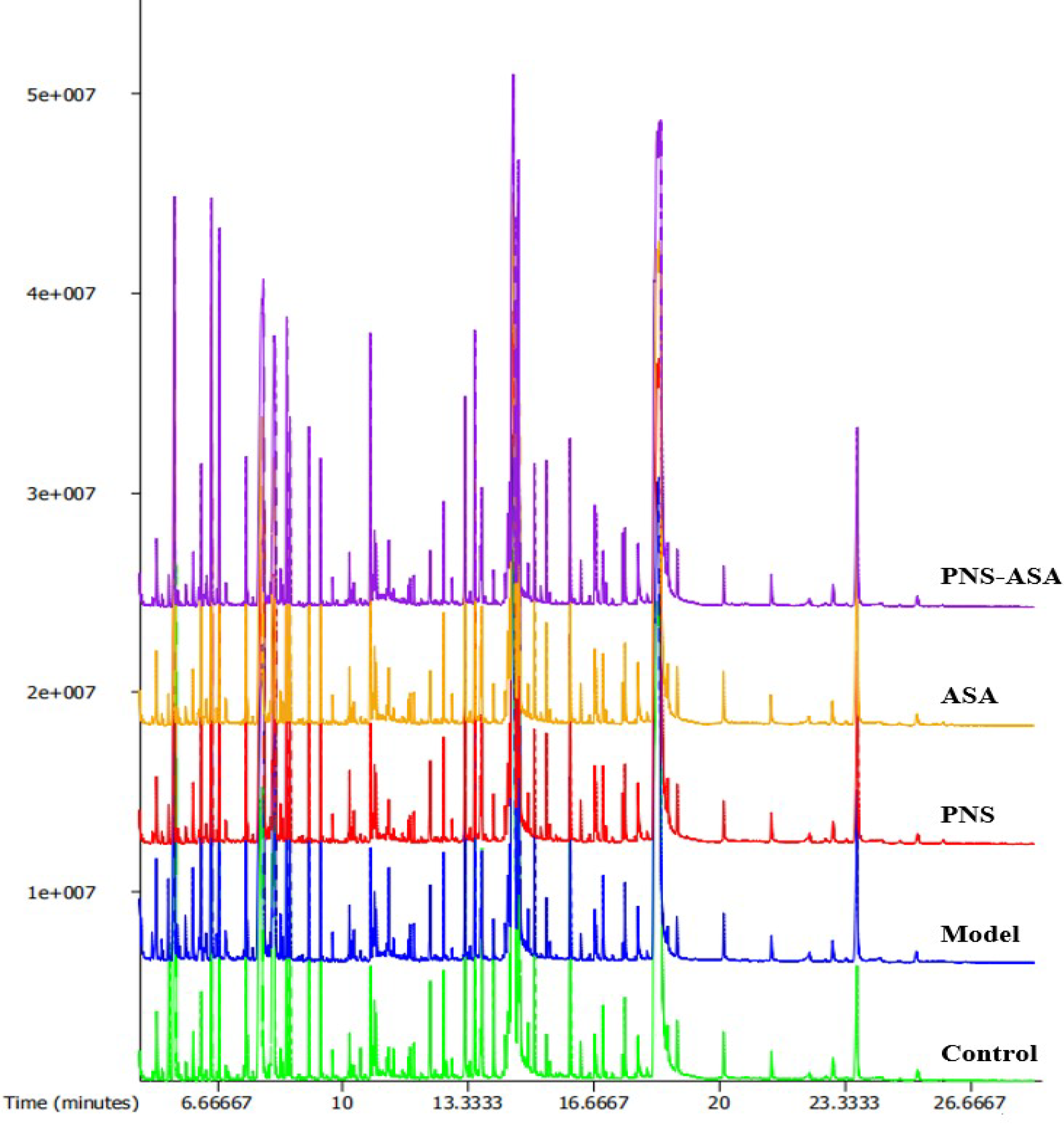

Supplement: Supplementary file 2 [file Image2.TIF]

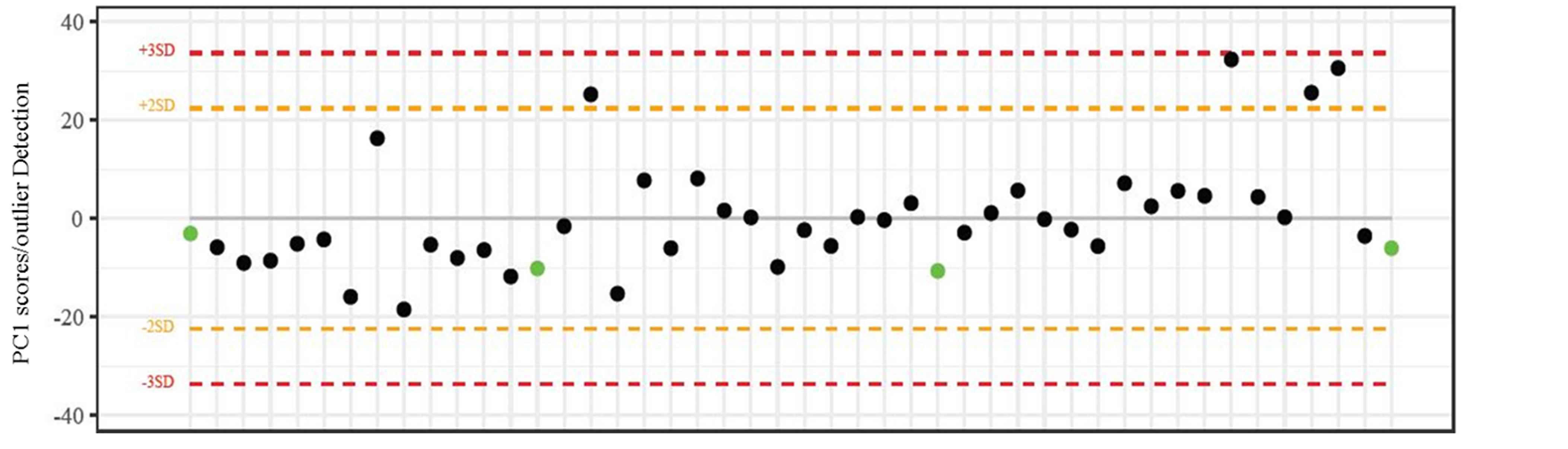

Supplement: Supplementary file 3 [file Image1.TIF]
