# Supplementary material for: Comparative Analysis of Compatibility Influence on Invigorating Blood Circulation for Combined Use of Panax Notoginseng Saponins and Aspirin Using Metabolomics Approach
Source: Front Pharmacol. 2021 Apr 30;12:544002. doi: 10.3389/fphar.2021.544002 (PMC8120290; doi:10.3389/fphar.2021.544002)
Supplement: Supplementary file 4 [file DataSheet1.PDF]

Figure S1

Chart of sample quality control. Green dots and black dots denote quality control samples and actual samples, respectively. The scores that exceed ± 3 standard deviations are generally considered as the risk of outliers.

Figure S2

Typical GC-TOF/MS total ion current chromatograms of rat plasma from the control, model, PNS, ASA, and PNS-ASA groups.

Figure S3

Orthogonal partial least squares discriminant analysis (OPLS-DA) based on acute blood stasis in rats. OPLS-DA scatter plots: (A) the control group versus the model group (R^2 = 0.982; Q^2 = 0.516); (C) the model group versus the PNS group (R^2 = 0.964; Q^2 = 0.454); (E) the model group versus the ASA group (R^2 = 0.904; Q^2 = 0.568); (G) the model group versus the combined use group (R^2 = 0.976; Q^2 = 0.680). Permutation validation plots: (B) the control group versus the model group; (D) the model group versus the ASA group; (F) the model group versus the ASA group; (H) the model group versus the combined use group.

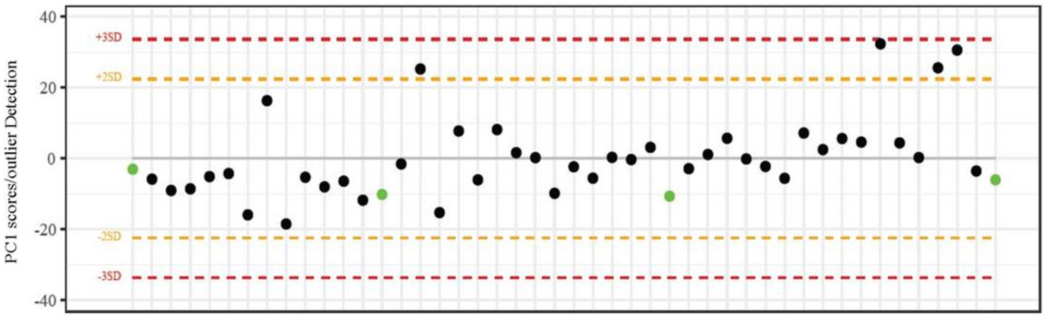

Figure S1

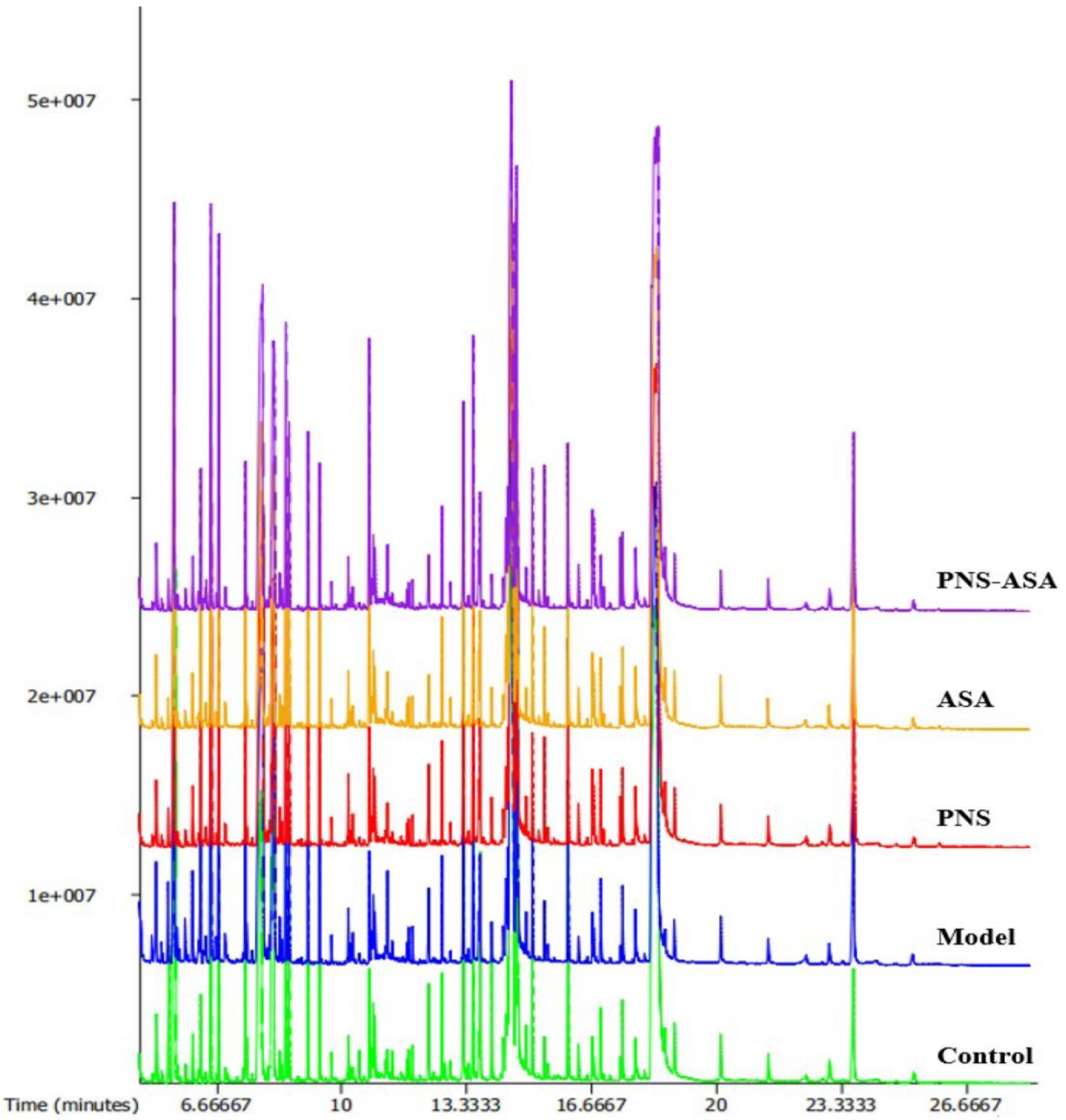

5e+007
4e+007
3e+007
2e+007
1e+007
Time (minutes)
6.66667
10
13.3333
16.6667
20
23.3333
26.6667
PNS-ASA
ASA
PNS
Model
Control

Figure S2

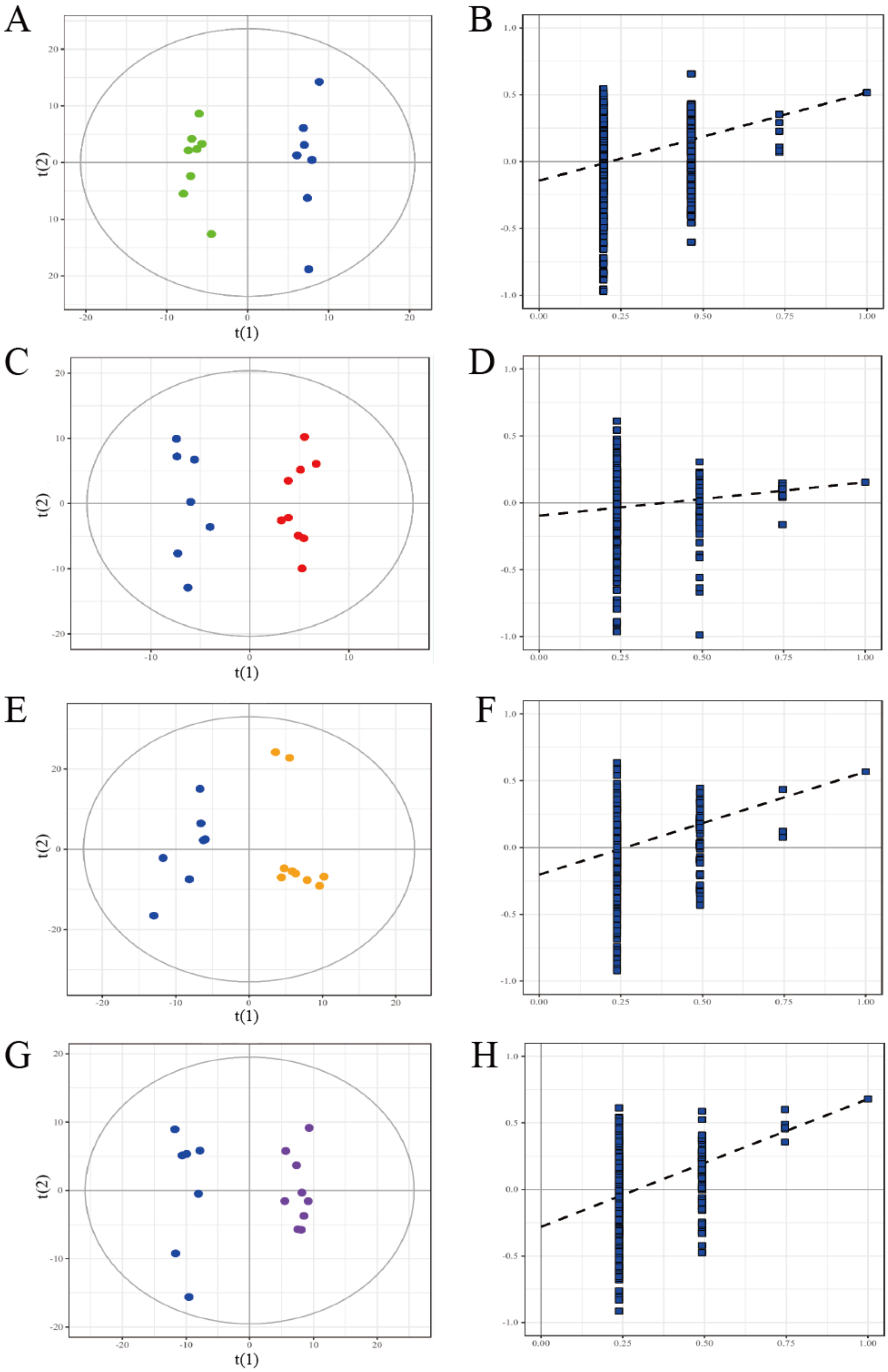

Figure S3
